# Crystal structure of 2-{[5-(methyl­sulfan­yl)-4-phenyl-4*H*-1,2,4-triazol-3-yl]meth­yl}benzo[*d*]thia­zole

**DOI:** 10.1107/S2056989023007041

**Published:** 2023-08-23

**Authors:** Rasha A. Azzam, Galal H. Elgemeie, Heba A. Elboshi, Peter G. Jones

**Affiliations:** a Chemistry Department, Faculty of Science, Helwan University, Cairo, Egypt; b Institut für Anorganische und Analytische Chemie, Technische Universität Braunschweig, Hagenring 30, D-38106 Braunschweig, Germany; Universität Greifswald, Germany

**Keywords:** crystal structure, triazole, benzo­thia­zole, thio­ether

## Abstract

In the structure of the title compound, C_17_H_14_N_4_O_2_, the triazole ring exhibits inter­planar angles of 63.86 (2) and 76.96 (2)° with the phenyl and benzo­thia­zole planes, respectively. The C—C—C angle at the methyl­ene group is rather wide at 114.28 (4)°. The packing involves three borderline C—H⋯N contacts, two of which combine to form layers parallel to *ac*, and a pairing of the triazole rings across an inversion centre [inter­planar distance of 3.1852 (2) Å].

## Abstract 2

In the crystal structure of the title compound, the triazole ring exhibits inter­planar angles of *ca* 64 and 77° with the phenyl and benzo­thia­zole planes, respectively. The packing involves three borderline C—H⋯N contacts and a pairing of the triazole rings across an inversion centre.

## 1. Chemical context

Benzo­thia­zoles and their derivatives are among the most important heterocyclic compounds in medicinal chemistry and are essential to many natural products and therapeutic preparations (Bonde *et al.*, 2015 ▸). The derivatives involve a wide range of structural variants (Rana *et al.*, 2008 ▸), and their pharmacological qualities are reflected in the extensive hunt for new therapeutically active compounds (Wang *et al.*, 2009 ▸), which represents a rapidly developing research area (Abdallah *et al.*, 2023*a* ▸,*b* ▸; Ammazzalorso *et al.*, 2020 ▸; Gill *et al.*, 2015 ▸). In particular, several substances based on benzo­thia­zole derivatives have been adapted and/or further developed for clinical practice to treat a wide range of diseases with great therapeutic efficacy (Huang *et al.*, 2009 ▸; Seenaiah *et al.*, 2014 ▸).

As part of our development of synthetic methods for the preparation of benzo­thia­zole-based heterocycles and other pharmaceutically inter­esting heterocycles (Ahmed *et al.*, 2022 ▸; Yakout *et al.*, 1999 ▸), we recently described the synthesis and biological activity of a series of 2-pyrimidyl- and 2-pyridyl-benzo­thia­zole derivatives with encouraging cytotoxic activity (Azzam *et al.* 2020*a* ▸,*b* ▸,*c* ▸, 2022*a* ▸,*b* ▸). 
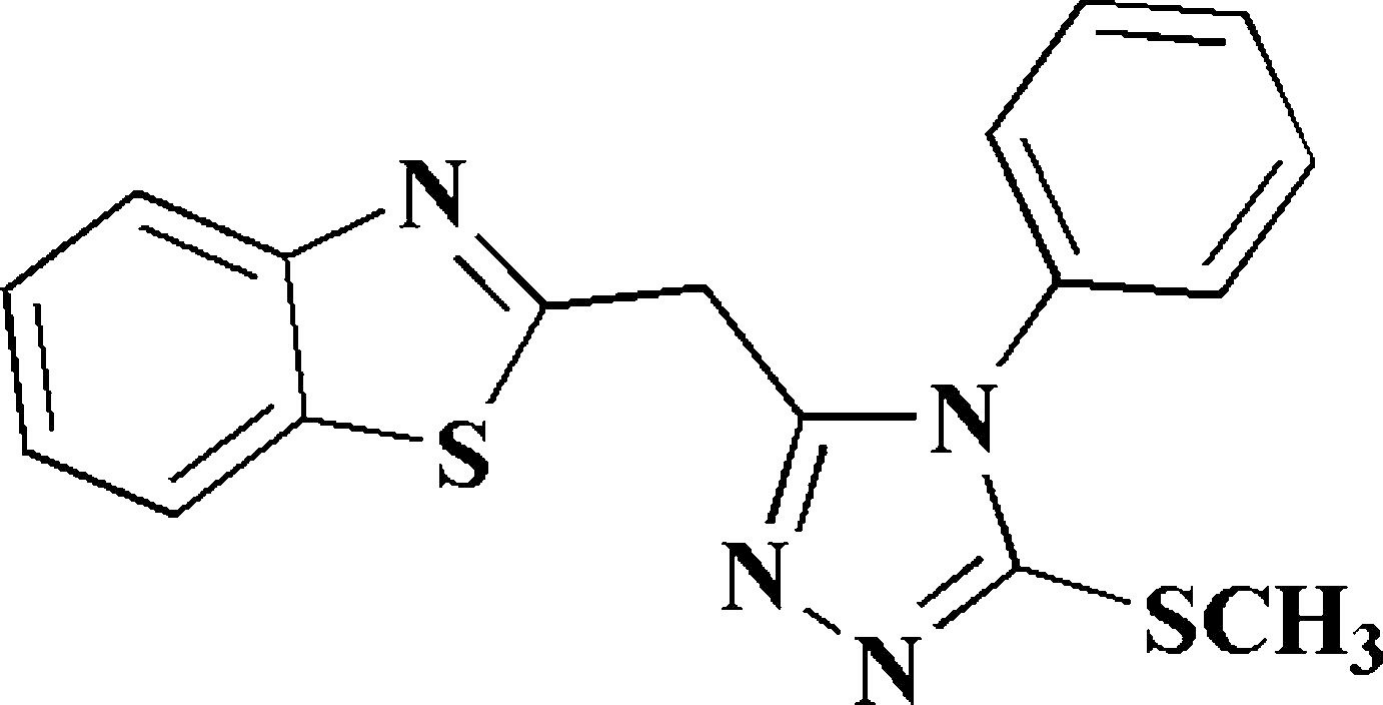


**Figure 1:**
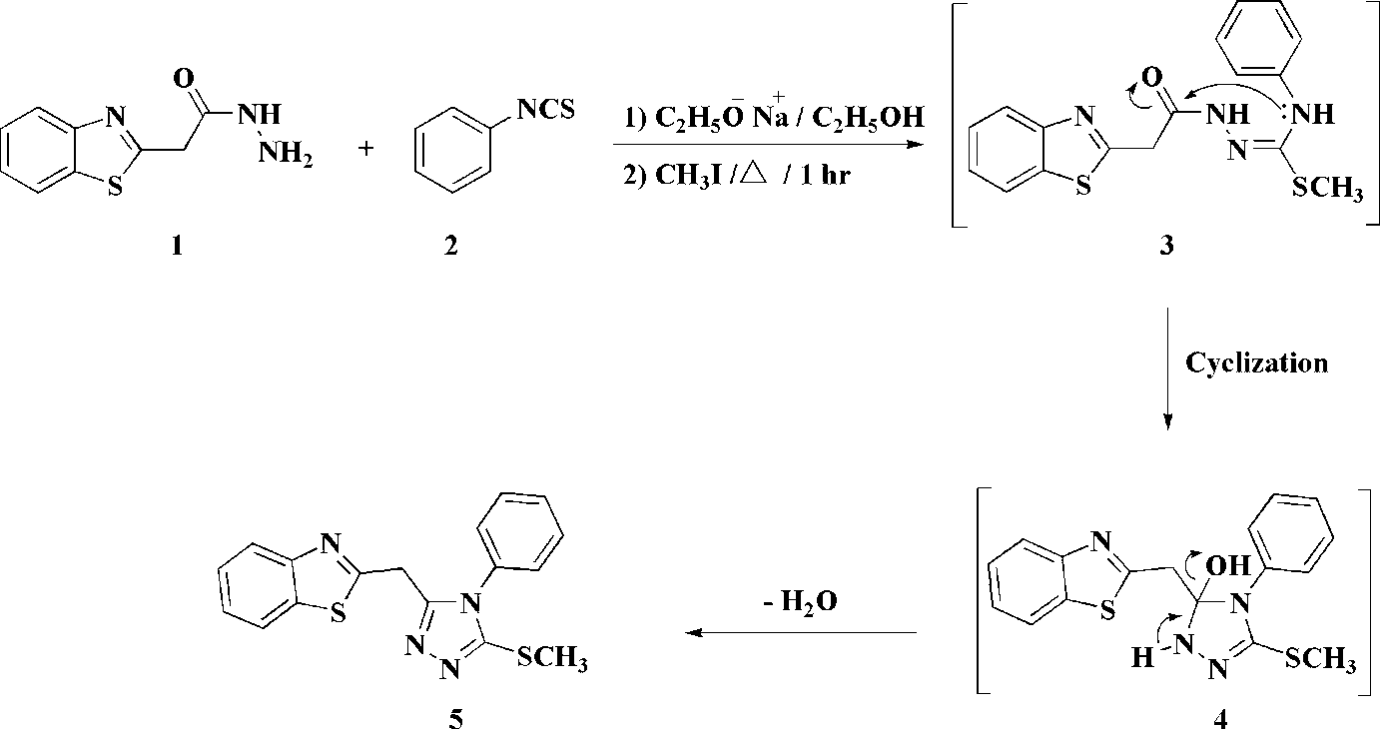
Reaction scheme for the synthesis of **5**.

As a continuation of this programme, related to our recent results (Elgemeie *et al.*, 2020 ▸, 2022 ▸; Metwally *et al.*, 2022*a* ▸,*b* ▸), the purpose of the present study was to design and synthesize benzo­thia­zolyl-triazole hybrids. The synthesis of our target benzo­thia­zole-2-triazole derivative **5** was achieved by reacting the 2-benzo­thia­zolyl acetohydrazide **1** with phenyl iso­thio­cyanate **2** in the presence of sodium ethoxide, followed by addition of methyl iodide to give **5** in good yield (Fig. 1 ▸). The formation of **5** is assumed to proceed *via* initial formation of adduct **4**, with subsequent elimination of water. In order to establish the structure of the product unambiguously, its crystal structure was determined and is reported here.

## 2. Structural commentary

**Figure 2:**
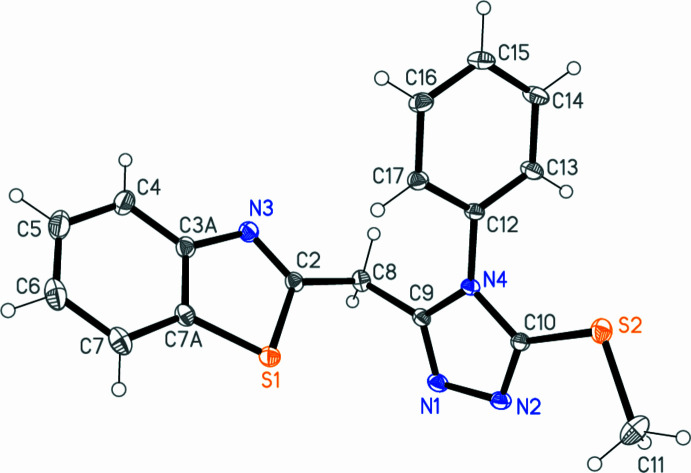
The mol­ecule of compound **5** in the crystal. Ellipsoids represent 50% probability levels.

The structure of compound **5** is shown in Fig. 2 ▸. Bond lengths and angles may be generally regarded as normal; *e.g*. the two S2—C bond lengths differ appreciably, reflecting the different hybridizations of C10 and C11. One exception may be the angle C2—C8—C9 at the methyl­ene group, which is rather wide at 114.28 (4)° (see below). A selection, mostly involving the heteroatoms, is presented in Table 1 ▸. The triazole ring subtends inter­planar angles of 63.86 (2) and 76.96 (2)° with the phenyl and benzo­thia­zole planes, respectively. The intra­molecular distance S1⋯N1 is 3.4819 (5) Å, far too long to represent any significant inter­action, in contrast to the value of 2.7570 (8) Å that we recently observed for the intra­molecular S⋯N_imine_ contact in *N*-[3-(benzo[d]thia­zol-2-yl)-6-bromo-2*H*-chromen-2-yl­idene]-4-methyl­benzenamine (Abdallah *et al.*, 2023*a* ▸).

## 3. Supra­molecular features

**Figure 3:**
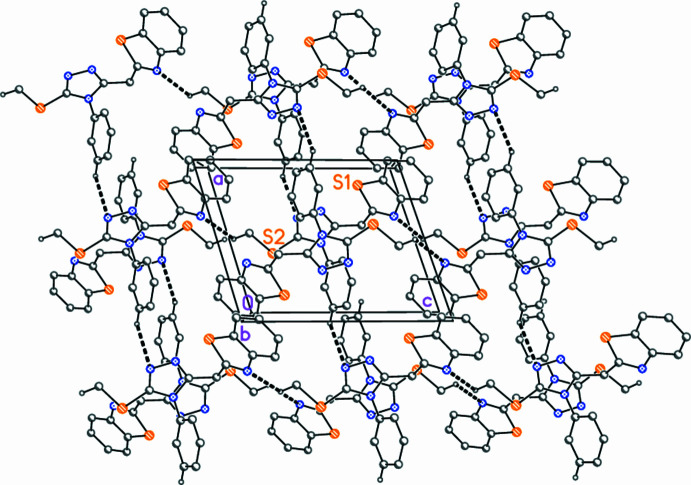
Packing diagram of compound **5**, showing the layer structure parallel to *ac* in the region *y* ≃ 0.25. Thick dashed bonds represent ‘weak’ C—H⋯N hydrogen bonds. The labelled atoms indicate the asymmetric unit.

The mol­ecular packing displays few significant features. There are three borderline C—H⋯N inter­actions (Table 2 ▸), two of which (the first and third in Table 2 ▸) connect the mol­ecules by translation to form thick layers parallel to the *ac* plane (Fig. 3 ▸). The triazole rings are associated in pairs (presumably representing a π–π inter­action) *via* the operator 1 − *x*, −*y*, 1 − *z*, with inter­centroid, inter­planar and offset distances of 3.3222 (3), 3.1852 (2) and 0.94 Å, respectively. This feature is reinforced by the other C—H⋯N inter­action, which involves the same operator.

## 4. Database survey

The searches employed the routine ConQuest (Bruno *et al.*, 2002 ▸), part of Version 2022.3.0 of the Cambridge Database (Groom *et al.*, 2016 ▸).

Only one other structure containing both a triazole and a benzo[*d*]thia­zole ring system was found, namely 2-(6-phenyl-7*H*-1,2,4-triazolo[3,4-*b*]-[1,3,4]thia­diazin-3-yl)-1,3-benzo­thia­zole (refcode AZUYEU; Abdel-Aziz *et al.*, 2011 ▸). This, however, contains a further heterocycle fused to the triazole ring.

To see if the C—C—C angle at the methyl­ene group of **5** is unusually wide, a search was performed for all structures with two five-membered rings connected across a methyl­ene group; the one restriction was that both of the outer carbon atoms should be three-coordinated. This led (excluding a few clear outliers) to 445 values in the range 106–122°, with a mean value of 114 (5)°. However, restricting one ring to be a C2-substituted thia­zole gave only three hits, with four values of 109.6–112.9° for the angle at the methyl­ene groups. These all involved two planar ring systems of the benzo[*d*]thia­zole type, but with different heteroatoms in some cases (HANSIB and HANSOH, Dauer *et al.*, 2017 ▸; KONTAK, Dauer & Stalke, 2014 ▸).

## 5. Synthesis and crystallization

A mixture of 2-benzo­thia­zolyl acetohydrazide **1** (0.01 mol) and phenyl iso­thio­cyanate **2** (0.01 mol) was stirred for 30 min in ethanol (25 mL) in the presence of sodium ethoxide (0.01 mol). After cooling, methyl iodide (0.015 mol) was added. The reaction mixture was stirred for 30 min at room temperature, then refluxed for 1 h. The resulting precipitate was filtered off, washed with water, dried, and recrystallized from ethanol. The title compound was isolated as a white solid; yield 75%; m.p. 429 K; IR (KBr, cm^−1^): *ν* 3053 (Ar—CH), 2928 (aliphatic H), 1594 (C=N); ^1^H NMR (400 MHz, DMSO-*d*_6_): δ 2.60 (*s*, 3H, SCH_3_), 4.57 (*s*, 2H, CH_2_), 7.36–7.50 (*m*, 7H, 5 Ar-H and 2 benzo­thia­zole-H), 7.89 (*d*, *J* = 8.0 Hz, 1H, benzo­thia­zole-H), 8.01 (*d*, *J* = 8.0 Hz, 1H, benzo­thia­zole-H); Analysis calculated for C_17_H_14_N_4_S_2_ (338.45): C 60.33, H 4.17, N 16.55, S 18.95. Found C 60.66; H 4.15; N 16.40; S 18.90%.

## 6. Refinement

Crystal data, data collection and structure refinement details are summarized in Table 3 ▸. The methyl group was included as an idealized rigid group allowed to rotate but not tip (C—H = 0.98 Å, H—C—H = 109.5°). Other hydrogen atoms were included using a riding model starting from calculated positions (C—H_aromatic_ = 0.95 Å, C—H_methyl­ene_ = 0.99 Å). The *U*(H) values were fixed at 1.5 × *U*_eq_ of the parent carbon atoms for the methyl group and 1.2 × *U*_eq_ for other hydrogens.

## Supplementary Material

Crystal structure: contains datablock(s) I, global. DOI: 10.1107/S2056989023007041/yz2038sup1.cif

Structure factors: contains datablock(s) I. DOI: 10.1107/S2056989023007041/yz2038Isup2.hkl

Supporting information file. DOI: 10.1107/S2056989023007041/yz2038Isup3.cml

CCDC reference: 2287438

Additional supporting information:  crystallographic information; 3D view; checkCIF report

## Figures and Tables

**Table 1:** Selected geometric parameters (Å, °)

S1—C7*A*	1.7334 (5)	N2—C10	1.3150 (6)
S1—C2	1.7503 (5)	N3—C2	1.2973 (6)
S2—C10	1.7418 (5)	N3—C3*A*	1.3905 (7)
S2—C11	1.8063 (6)	N4—C10	1.3716 (6)
N1—C9	1.3094 (6)	N4—C9	1.3754 (6)
N1—N2	1.3968 (6)		
			
C7*A*—S1—C2	88.86 (2)	C10—N4—C9	104.30 (4)
C10—S2—C11	98.33 (3)	N3—C2—S1	116.30 (4)
C9—N1—N2	107.60 (4)	C9—C8—C2	114.28 (4)
C10—N2—N1	106.63 (4)	N1—C9—N4	110.51 (4)
C2—N3—C3*A*	110.40 (4)	N2—C10—N4	110.97 (4)

**Table 2:** Hydrogen-bond geometry (Å, °)

*D*—H⋯*A*	*D*—H	H⋯*A*	*D*⋯*A*	*D*—H⋯*A*
C11—H11*A*⋯N3^i^	0.98	2.68	3.3610 (8)	127
C13—H13⋯N1^ii^	0.95	2.68	3.3609 (6)	129
C14—H14⋯N2^iii^	0.95	2.67	3.3431 (6)	129

Symmetry codes: (i) 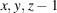 ; (ii) 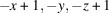 ; (iii) 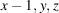 .

**Table 3:** Experimental details

Crystal data	
Chemical formula	C_17_H_14_N_4_S_2_
*M*_r_	338.44
Crystal system, space group	Triclinic, *P* 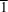
Temperature (K)	100
*a*, *b*, *c* (Å)	8.9714 (2), 9.3564 (3), 10.4969 (2)
α, β, γ (°)	94.088 (2), 105.954 (2), 107.393 (2)
*V* (Å^3^)	797.05 (3)
*Z*	2
Radiation type	Mo *K*α
μ (mm^−1^)	0.34
Crystal size (mm)	0.17 × 0.12 × 0.10

Data collection	
Diffractometer	XtaLAB Synergy
Absorption correction	Multi-scan (*CrysAlis PRO*; Rigaku OD, 2021 ▸)
*T*_min_, *T*_max_	0.859, 1.000
No. of measured, independent and observed [*I* > 2σ(*I*)] reflections	102462, 10466, 9289
*R*_int_	0.033
(sin θ/λ)_max_ (Å^−1^)	0.927

Refinement	
*R*[*F* ^2^ > 2σ(*F* ^2^)], *wR*(*F* ^2^), *S*	0.028, 0.086, 1.04
No. of reflections	10466
No. of parameters	209
H-atom treatment	H-atom parameters constrained
Δρ_max_, Δρ_min_ (e Å^−3^)	0.66, −0.25

Computer programs: *CrysAlis PRO* (Rigaku OD, 2021 ▸), *SHELXT* (Sheldrick, 2015*a* ▸), *SHELXL2019/3* (Sheldrick, 2015*b* ▸) and *XP* (Siemens, 1994 ▸).

## supplementary crystallographic information

### Crystal data

**Table:**

C_17_H_14_N_4_S_2_	*Z* = 2
*M**_r_* = 338.44	*F*(000) = 352
Triclinic, *P*1	*D*_x_ = 1.410 Mg m^−^^3^
*a* = 8.9714 (2) Å	Mo *K*α radiation, λ = 0.71073 Å
*b* = 9.3564 (3) Å	Cell parameters from 69237 reflections
*c* = 10.4969 (2) Å	θ = 2.3–41.4°
α = 94.088 (2)°	µ = 0.34 mm^−^^1^
β = 105.954 (2)°	*T* = 100 K
γ = 107.393 (2)°	Tablet, colourless
*V* = 797.05 (3) Å^3^	0.17 × 0.12 × 0.10 mm

### Data collection

**Table:**

XtaLAB Synergy diffractometer	10466 independent reflections
Radiation source: micro-focus sealed X-ray tube, PhotonJet (Mo) X-ray Source	9289 reflections with *I* > 2σ(*I*)
Mirror monochromator	*R*_int_ = 0.033
Detector resolution: 10.0000 pixels mm^-1^	θ_max_ = 41.2°, θ_min_ = 2.1°
ω scans	*h* = −16→16
Absorption correction: multi-scan (CrysAlisPro; Rigaku OD, 2021)	*k* = −17→17
*T*_min_ = 0.859, *T*_max_ = 1.000	*l* = −19→19
102462 measured reflections	

### Refinement

**Table:**

Refinement on *F*^2^	Primary atom site location: dual
Least-squares matrix: full	Hydrogen site location: inferred from neighbouring sites
*R*[*F*^2^ > 2σ(*F*^2^)] = 0.028	H-atom parameters constrained
*wR*(*F*^2^) = 0.086	*w* = 1/[σ^2^(*F*_o_^2^) + (0.0528*P*)^2^ + 0.0903*P*] where *P* = (*F*_o_^2^ + 2*F*_c_^2^)/3
*S* = 1.04	(Δ/σ)_max_ = 0.001
10466 reflections	Δρ_max_ = 0.66 e Å^−^^3^
209 parameters	Δρ_min_ = −0.25 e Å^−^^3^
0 restraints	

### Special details

**Table:**

Geometry. All esds (except the esd in the dihedral angle between two l.s. planes) are estimated using the full covariance matrix. The cell esds are taken into account individually in the estimation of esds in distances, angles and torsion angles; correlations between esds in cell parameters are only used when they are defined by crystal symmetry. An approximate (isotropic) treatment of cell esds is used for estimating esds involving l.s. planes. Least-squares planes (x,y,z in crystal coordinates) and deviations from them (* indicates atom used to define plane) - 0.6560 (0.0019) x - 4.9080 (0.0018) y + 9.3027 (0.0011) z = 3.1483 (0.0010) * -0.0006 (0.0003) C12 * 0.0030 (0.0004) C13 * -0.0029 (0.0004) C14 * 0.0003 (0.0004) C15 * 0.0021 (0.0004) C16 * -0.0019 (0.0003) C17 0.0217 (0.0007) N4 Rms deviation of fitted atoms = 0.0021 2.6765 (0.0019) x + 7.5840 (0.0013) y - 0.9757 (0.0024) z = 2.4430 (0.0013) Angle to previous plane (with approximate esd) = 63.863 ( 0.019 ) * -0.0001 (0.0003) N1 * 0.0018 (0.0003) N2 * 0.0025 (0.0002) N4 * -0.0014 (0.0003) C9 * -0.0027 (0.0003) C10 -0.0058 (0.0008) C8 -0.0865 (0.0007) C12 0.0346 (0.0007) S2 Rms deviation of fitted atoms = 0.0019 5.3893 (0.0012) x - 6.3624 (0.0008) y + 5.1564 (0.0013) z = 5.5973 (0.0010) Angle to previous plane (with approximate esd) = 76.957 ( 0.014 ) * -0.0079 (0.0003) S1 * 0.0083 (0.0003) C2 * -0.0011 (0.0004) N3 * 0.0009 (0.0004) C3A * -0.0081 (0.0005) C4 * 0.0025 (0.0005) C5 * 0.0038 (0.0005) C6 * -0.0013 (0.0004) C7 * 0.0028 (0.0004) C7A 0.0979 (0.0006) C8 Rms deviation of fitted atoms = 0.0050

### Fractional atomic coordinates and isotropic or equivalent isotropic displacement parameters (Å^2^)

**Table:**

	*x*	*y*	*z*	*U*_iso_*/*U*_eq_	
S1	0.86054 (2)	0.48129 (2)	0.77843 (2)	0.01557 (3)	
S2	0.40521 (2)	0.21180 (2)	0.21857 (2)	0.01692 (3)	
N1	0.69326 (5)	0.14743 (5)	0.54398 (4)	0.01461 (6)	
N2	0.64593 (5)	0.14656 (5)	0.40548 (4)	0.01439 (6)	
N3	0.65416 (5)	0.41599 (5)	0.91486 (4)	0.01501 (6)	
N4	0.47354 (4)	0.21828 (4)	0.48932 (4)	0.01123 (5)	
C2	0.68997 (5)	0.36293 (5)	0.81379 (5)	0.01319 (6)	
C3A	0.76637 (6)	0.55937 (6)	0.97489 (5)	0.01493 (7)	
C4	0.76214 (7)	0.64760 (7)	1.08644 (6)	0.02094 (9)	
H4	0.678550	0.611765	1.127062	0.025*	
C5	0.88296 (8)	0.78864 (7)	1.13624 (6)	0.02484 (10)	
H5	0.882744	0.849356	1.212644	0.030*	
C6	1.00541 (8)	0.84304 (7)	1.07564 (7)	0.02362 (10)	
H6	1.086509	0.940173	1.111582	0.028*	
C7	1.01035 (7)	0.75745 (6)	0.96388 (6)	0.02010 (9)	
H7	1.092893	0.794830	0.922535	0.024*	
C7A	0.88963 (6)	0.61445 (5)	0.91440 (5)	0.01491 (7)	
C8	0.59619 (6)	0.20550 (5)	0.73494 (5)	0.01490 (7)	
H8A	0.647569	0.133827	0.778009	0.018*	
H8B	0.482421	0.175752	0.739460	0.018*	
C9	0.58961 (5)	0.18991 (5)	0.59118 (5)	0.01228 (6)	
C10	0.51471 (5)	0.18851 (5)	0.37612 (5)	0.01212 (6)	
C11	0.54143 (10)	0.18497 (8)	0.12746 (7)	0.02628 (11)	
H11A	0.500730	0.200775	0.034712	0.039*	
H11B	0.545540	0.081470	0.127332	0.039*	
H11C	0.651920	0.258121	0.170908	0.039*	
C12	0.33320 (5)	0.25713 (5)	0.49752 (5)	0.01169 (6)	
C13	0.17638 (6)	0.15400 (5)	0.43243 (6)	0.01604 (7)	
H13	0.162422	0.059676	0.382357	0.019*	
C14	0.04025 (6)	0.19174 (6)	0.44212 (6)	0.01850 (8)	
H14	−0.067511	0.123172	0.397683	0.022*	
C15	0.06206 (6)	0.32983 (6)	0.51686 (6)	0.01743 (8)	
H15	−0.030950	0.354811	0.523584	0.021*	
C16	0.21950 (6)	0.43126 (6)	0.58166 (5)	0.01627 (7)	
H16	0.233522	0.525098	0.632575	0.020*	
C17	0.35680 (6)	0.39572 (5)	0.57216 (5)	0.01364 (7)	
H17	0.464502	0.464813	0.615821	0.016*	

### Atomic displacement parameters (Å^2^)

**Table:**

	*U*^11^	*U*^22^	*U*^33^	*U*^12^	*U*^13^	*U*^23^
S1	0.01412 (5)	0.01676 (5)	0.01501 (5)	0.00285 (4)	0.00577 (4)	0.00253 (4)
S2	0.01817 (5)	0.01874 (5)	0.01337 (5)	0.00661 (4)	0.00344 (4)	0.00346 (4)
N1	0.01107 (13)	0.01546 (14)	0.01739 (16)	0.00585 (11)	0.00320 (12)	0.00174 (12)
N2	0.01163 (13)	0.01526 (14)	0.01706 (16)	0.00536 (11)	0.00506 (12)	0.00130 (12)
N3	0.01422 (14)	0.01645 (15)	0.01332 (15)	0.00367 (12)	0.00428 (12)	0.00227 (12)
N4	0.00944 (12)	0.01239 (13)	0.01242 (14)	0.00476 (10)	0.00313 (10)	0.00170 (10)
C2	0.01226 (15)	0.01421 (15)	0.01189 (15)	0.00350 (12)	0.00266 (12)	0.00293 (12)
C3A	0.01476 (16)	0.01592 (16)	0.01294 (16)	0.00514 (13)	0.00257 (13)	0.00182 (13)
C4	0.0226 (2)	0.0228 (2)	0.01640 (19)	0.00840 (18)	0.00479 (17)	−0.00129 (16)
C5	0.0265 (2)	0.0221 (2)	0.0212 (2)	0.00883 (19)	0.00129 (19)	−0.00446 (18)
C6	0.0222 (2)	0.01629 (19)	0.0242 (2)	0.00439 (17)	−0.00227 (18)	−0.00112 (17)
C7	0.01646 (18)	0.01581 (18)	0.0226 (2)	0.00197 (14)	0.00139 (16)	0.00295 (16)
C7A	0.01367 (16)	0.01456 (16)	0.01459 (17)	0.00406 (13)	0.00200 (13)	0.00291 (13)
C8	0.01522 (16)	0.01389 (15)	0.01334 (16)	0.00301 (13)	0.00283 (13)	0.00258 (12)
C9	0.01024 (14)	0.01221 (14)	0.01353 (16)	0.00399 (11)	0.00216 (12)	0.00169 (12)
C10	0.01095 (14)	0.01190 (14)	0.01336 (16)	0.00363 (11)	0.00398 (12)	0.00119 (12)
C11	0.0363 (3)	0.0289 (3)	0.0200 (2)	0.0127 (2)	0.0159 (2)	0.00653 (19)
C12	0.00982 (13)	0.01180 (14)	0.01430 (16)	0.00444 (11)	0.00418 (12)	0.00211 (12)
C13	0.01030 (14)	0.01344 (16)	0.0229 (2)	0.00391 (12)	0.00377 (14)	−0.00030 (14)
C14	0.01035 (15)	0.01583 (17)	0.0290 (2)	0.00453 (13)	0.00582 (15)	0.00189 (16)
C15	0.01381 (16)	0.01674 (17)	0.0259 (2)	0.00773 (14)	0.00937 (16)	0.00506 (15)
C16	0.01628 (17)	0.01471 (16)	0.0206 (2)	0.00720 (14)	0.00803 (15)	0.00189 (14)
C17	0.01255 (15)	0.01250 (15)	0.01612 (17)	0.00444 (12)	0.00489 (13)	0.00105 (12)

### Geometric parameters (Å, º)

**Table:**

S1—C7A	1.7334 (5)	C12—C17	1.3928 (6)
S1—C2	1.7503 (5)	C12—C13	1.3940 (6)
S2—C10	1.7418 (5)	C13—C14	1.3955 (7)
S2—C11	1.8063 (6)	C14—C15	1.3939 (7)
N1—C9	1.3094 (6)	C15—C16	1.3917 (7)
N1—N2	1.3968 (6)	C16—C17	1.3948 (6)
N2—C10	1.3150 (6)	C4—H4	0.9500
N3—C2	1.2973 (6)	C5—H5	0.9500
N3—C3A	1.3905 (7)	C6—H6	0.9500
N4—C10	1.3716 (6)	C7—H7	0.9500
N4—C9	1.3754 (6)	C8—H8A	0.9900
N4—C12	1.4330 (5)	C8—H8B	0.9900
C2—C8	1.5050 (7)	C11—H11A	0.9800
C3A—C4	1.3997 (7)	C11—H11B	0.9800
C3A—C7A	1.4084 (7)	C11—H11C	0.9800
C4—C5	1.3873 (9)	C13—H13	0.9500
C5—C6	1.4023 (10)	C14—H14	0.9500
C6—C7	1.3912 (9)	C15—H15	0.9500
C7—C7A	1.3994 (7)	C16—H16	0.9500
C8—C9	1.4887 (7)	C17—H17	0.9500
			
C7A—S1—C2	88.86 (2)	C16—C15—C14	120.28 (4)
C10—S2—C11	98.33 (3)	C15—C16—C17	120.28 (4)
C9—N1—N2	107.60 (4)	C12—C17—C16	118.83 (4)
C10—N2—N1	106.63 (4)	C5—C4—H4	120.8
C2—N3—C3A	110.40 (4)	C3A—C4—H4	120.8
C10—N4—C9	104.30 (4)	C4—C5—H5	119.5
C10—N4—C12	127.66 (4)	C6—C5—H5	119.5
C9—N4—C12	127.84 (4)	C7—C6—H6	119.4
N3—C2—C8	122.28 (4)	C5—C6—H6	119.4
N3—C2—S1	116.30 (4)	C6—C7—H7	121.1
C8—C2—S1	121.35 (3)	C7A—C7—H7	121.1
N3—C3A—C4	124.68 (5)	C9—C8—H8A	108.7
N3—C3A—C7A	115.00 (4)	C2—C8—H8A	108.7
C4—C3A—C7A	120.32 (5)	C9—C8—H8B	108.7
C5—C4—C3A	118.40 (6)	C2—C8—H8B	108.7
C4—C5—C6	121.08 (6)	H8A—C8—H8B	107.6
C7—C6—C5	121.24 (5)	S2—C11—H11A	109.5
C6—C7—C7A	117.76 (5)	S2—C11—H11B	109.5
C7—C7A—C3A	121.20 (5)	H11A—C11—H11B	109.5
C7—C7A—S1	129.37 (4)	S2—C11—H11C	109.5
C3A—C7A—S1	109.43 (3)	H11A—C11—H11C	109.5
C9—C8—C2	114.28 (4)	H11B—C11—H11C	109.5
N1—C9—N4	110.51 (4)	C12—C13—H13	120.6
N1—C9—C8	124.86 (4)	C14—C13—H13	120.6
N4—C9—C8	124.63 (4)	C15—C14—H14	119.9
N2—C10—N4	110.97 (4)	C13—C14—H14	119.9
N2—C10—S2	126.93 (4)	C16—C15—H15	119.9
N4—C10—S2	122.07 (3)	C14—C15—H15	119.9
C17—C12—C13	121.63 (4)	C15—C16—H16	119.9
C17—C12—N4	119.25 (4)	C17—C16—H16	119.9
C13—C12—N4	119.12 (4)	C12—C17—H17	120.6
C12—C13—C14	118.84 (4)	C16—C17—H17	120.6
C15—C14—C13	120.14 (5)		
			
C9—N1—N2—C10	−0.19 (5)	C12—N4—C9—N1	175.49 (4)
C3A—N3—C2—C8	175.98 (4)	C10—N4—C9—C8	−179.64 (4)
C3A—N3—C2—S1	−1.04 (5)	C12—N4—C9—C8	−4.54 (7)
C7A—S1—C2—N3	1.07 (4)	C2—C8—C9—N1	93.15 (6)
C7A—S1—C2—C8	−175.98 (4)	C2—C8—C9—N4	−86.82 (5)
C2—N3—C3A—C4	179.97 (5)	N1—N2—C10—N4	0.44 (5)
C2—N3—C3A—C7A	0.43 (6)	N1—N2—C10—S2	178.46 (3)
N3—C3A—C4—C5	179.66 (5)	C9—N4—C10—N2	−0.51 (5)
C7A—C3A—C4—C5	−0.82 (8)	C12—N4—C10—N2	−175.63 (4)
C3A—C4—C5—C6	0.86 (9)	C9—N4—C10—S2	−178.64 (3)
C4—C5—C6—C7	−0.20 (10)	C12—N4—C10—S2	6.24 (6)
C5—C6—C7—C7A	−0.49 (9)	C11—S2—C10—N2	−6.70 (5)
C6—C7—C7A—C3A	0.52 (8)	C11—S2—C10—N4	171.12 (4)
C6—C7—C7A—S1	179.73 (4)	C10—N4—C12—C17	−119.71 (5)
N3—C3A—C7A—C7	179.70 (5)	C9—N4—C12—C17	66.28 (6)
C4—C3A—C7A—C7	0.13 (8)	C10—N4—C12—C13	61.39 (6)
N3—C3A—C7A—S1	0.35 (5)	C9—N4—C12—C13	−112.62 (5)
C4—C3A—C7A—S1	−179.22 (4)	C17—C12—C13—C14	0.38 (8)
C2—S1—C7A—C7	179.99 (5)	N4—C12—C13—C14	179.25 (5)
C2—S1—C7A—C3A	−0.73 (4)	C12—C13—C14—C15	−0.59 (8)
N3—C2—C8—C9	149.86 (5)	C13—C14—C15—C16	0.35 (9)
S1—C2—C8—C9	−33.27 (6)	C14—C15—C16—C17	0.12 (8)
N2—N1—C9—N4	−0.13 (5)	C13—C12—C17—C16	0.08 (7)
N2—N1—C9—C8	179.90 (4)	N4—C12—C17—C16	−178.79 (4)
C10—N4—C9—N1	0.39 (5)	C15—C16—C17—C12	−0.33 (7)

### Hydrogen-bond geometry (Å, º)

**Table:**

*D*—H···*A*	*D*—H	H···*A*	*D*···*A*	*D*—H···*A*
C11—H11*A*···N3^i^	0.98	2.68	3.3610 (8)	127
C13—H13···N1^ii^	0.95	2.68	3.3609 (6)	129
C14—H14···N2^iii^	0.95	2.67	3.3431 (6)	129

Symmetry codes: (i) *x*, *y*, *z*−1; (ii) −*x*+1, −*y*, −*z*+1; (iii) *x*−1, *y*, *z*.
